# Frail-VIG index: a concise frailty evaluation tool for rapid geriatric assessment

**DOI:** 10.1186/s12877-018-0718-2

**Published:** 2018-01-26

**Authors:** Jordi Amblàs-Novellas, Joan Carles Martori, Joan Espaulella, Ramon Oller, Núria Molist-Brunet, Marco Inzitari, Roman Romero-Ortuno

**Affiliations:** 1 Geriatric and Palliative Care Department, Hospital Universitari de la Santa Creu / Hospital Universitari de Vic, Rambla Hospital 52, 08500 Vic, Barcelona Spain; 2 Department of Palliative Care, University of Vic / Central University of Catalonia, Barcelona, Spain; 3 Programme for the Prevention and Care of Patients with Chronic Conditions, Department of Health, Government of Catalonia, Barcelona, Spain; 4 Data Analysis and Modeling Research Group. Department of Economics and Business, University of Vic / Central University of Catalonia, Barcelona, Spain; 5 Parc Sanitari Pere Virgili. Universitat Autònoma de Barcelona, Barcelona, Spain; 6 Department of Medicine for the Elderly, Addenbrooke’s Hospital, Cambridge, UK; 7 Department of Public Health and Primary Care, Clinical Gerontology Unit, University of Cambridge, Cambridge, UK

**Keywords:** Frail elderly, Frailty index, Geriatric assessment, Multimorbidity, Mortality

## Abstract

**Background:**

Demographic changes have led to an increase in the number of elderly frail persons and, consequently, systematic geriatric assessment is more important than ever. Frailty Indexes (FI) may be particularly useful to discriminate between various degrees of frailty but are not routinely assessed due, at least in part, to the large number of deficits assessed (from 30 to 70). Therefore, we have developed a new, more concise FI for rapid geriatric assessment (RGA)—the Frail-VIG index (“VIG” is the Spanish/Catalan abbreviation for Comprehensive Geriatric Assessment), which contains 22 simple questions that assess 25 different deficits. Here we describe this FI and report its ability to predict mortality at 24 months.

**Methods:**

Prospective, observational, longitudinal study of geriatric patients followed for 24 months or until death. The study participants were patients (*n* = 590) admitted to the Acute Geriatric Unit at the at the University Hospital of Vic (Barcelona) during the year 2014. Participants were classified into one of seven groups based on their Frail-VIG score (0–0.15; 0.16–0.25; 0.26–0.35; 0.36–0.45; 0.46–0.55; 0.56–0.65; and 0.66–1). Survival curves for these groups were compared using the log-rank test. ROC curves were used to assess the index’s capacity to predict mortality at 24 months.

**Results:**

Mean (standard deviation) patient age was 86.4 (5.6) years. The 24-month mortality rate was 57.3% for the whole sample. Significant between-group (deceased vs. living) differences (*p* < 0.05) were observed for most index variables. Survival curves for the seven Frail-VIG groups differed significantly (X^2^ = 433.4, *p* < 0.001), with an area under the ROC curve (confidence interval) of 0.90 (0.88–0.92) at 12 months and 0.85 (0.82–0.88) at 24 months. Administration time for the Frail-VIG index ranged from 5 to 10 min.

**Conclusions:**

The Frail-VIG index, which requires less time to administer than previously validated FIs, presents a good discriminative capacity for the degree of frailty and a high predictive capacity for mortality in the present cohort. Although more research is needed to confirm the validity of this instrument in other populations and settings, the Frail-VIG may provide clinicians with a RGA method and also a reliable tool to assess frailty in routine practice.

**Electronic supplementary material:**

The online version of this article (10.1186/s12877-018-0718-2) contains supplementary material, which is available to authorized users.

## Background

Frailty is considered a clinical state in which the individual is more vulnerable to becoming dependent and/or in which there is a higher risk of death if exposed to a stressor [1]. Together with multimorbidity, frailty is one of the most common chronic conditions in the adult population [2], and both are closely associated with adverse health outcomes [3–5]. Despite controversies surrounding the optimum operational definition of frailty, there is a strong consensus among clinicians that frailty should be evaluated whenever feasible [6–8]. Two complementary approaches to assessing frailty that may be useful in different circumstances and for different purposes are Fried’s frailty syndrome approach [9, 10], which may be particularly useful for population screening to identify pre-disability states [11], and Rockwood’s deficit accumulation model [12], which views frailty as a continuum, and which may be particularly useful to discriminate between various degrees of frailty [13].

A wide variety of frailty indices (FIs) have been developed [3, 4, 12, 14, 15] -with the main differences among them being the number of domains [16] and deficits assessed (from 30 to 70 deficits) [12]. In most FIs, the probability of dying increases exponentially as the rate of deficit accumulation increases [14, 17–20]. Current FIs assess frailty on a scale ranging from 0 to 1, with scores from 0.2 [15] to 0.25 [21] indicating frailty. A score of 0.7 is usually considered the cut-off point to indicate that homeostasis has reached its limit and thus any additional deficits would likely result in death [16, 22]. The multidimensional nature of FIs is a characteristic that these instruments share with comprehensive geriatric assessment (CGA). Indeed, the close relation between FIs and CGA is not new: a study published in 2004 demonstrated that it was both clinically justified and operationally feasible to develop an FI based on geriatric assessment (FI-CGA) [23].

Despite the growing number of elderly persons with multimorbidity and frailty [24–26], which implies a need for healthcare providers to make geriatric assessment a routine part of healthcare delivery [27], the use of a complete CGA and/or FIs remain underutilized in many settings [28]. One of the main reasons for this lack of widespread adoption, particularly in the context of acute care hospitals [29–32], could be the amount of time needed to administer these instruments [14]. In this context, and given the importance of performing a situational diagnosis/assessment of the degree of frailty as the starting point for individualized decision-making [33], newer, more pragmatic and faster multidimensional tools are needed [34].

### Aims

Based on the multidimensional approach of the CGA and inspired by the simplicity and potential universal applicability of the Rapid Geriatric Assessment (RGA) [34], together with the known value of the FI methodology, we have developed a new FI called the “Frail-VIG index” (VIG is the Spanish/Catalan abbreviation for CGA). In the present observational study, we prospectively applied the Frail-VIG index to 590 patients admitted to the Acute Geriatric Unit (AGU) at the University Hospital of Vic (Barcelona, Spain) to determine the instrument’s capacity to predict mortality at 24 months.

## Methods

### Study design

This was a prospective, observational, longitudinal study.

### Context and participants

The study was conducted at the University Hospital of Vic (Barcelona; Spain), a 200-bed acute care hospital covering a population area of 156,000 inhabitants. All patients admitted to the AGU during the year 2014 were included. Admission criteria to the AGU were: 1) age ≥ 85 years; or 2) cognitive decline; or 3) the presence of advanced chronic conditions identified by the NECPAL (NECesidades PALiativas, in Spanish; Palliative Care Needs, in English) test [35], a validated tool for the early diagnosis of the need for palliative care among individuals with limited life expectancy. No exclusion criteria were defined.

### Design and evaluation of the frail-VIG index

To design the most pragmatic instrument possible, the Frail-VIG index was constructed using only variables recorded during the usual clinical evaluation process—as opposed to variables collected as part of an epidemiological study, which is how most other FIs were developed [12, 15, 18, 36, 37]. In our department, the original aim was to systematically perform a CGA for all patients; to achieve this, all health care professionals in the department agreed by consensus to create a checklist that included 40 multidimensional variables. Subsequently, given the need for a quantifiable RGA for decision-making at our acute care hospital [33], a proposal was made to reduce the number of variables, prioritizing those variables with the strongest prognostic capacity for mortality [38].

In the development of the Frail-VIG index, rather than comparing the index to a “gold standard” instrument, we sought to replicate the characteristics of a previously validated instrument [3, 4, 12, 14, 15] and then we evaluated its predictive ability [39]. Our development process focused on two main areas: 1) variable selection and construct development, and 2) demonstration of the predictive validity for mortality. The design of the Frail-VIG index has been previously described [40].

#### Variable selection and construct development

Published recommendations [15] suggest that all variables included in a FI must have the following characteristics: 1) prevalence of the variable must increase with age and be associated with health problems; 2) it should not saturate too early; 3) it should not be excessively prevalent (> 80% of individuals ≥ age 85) or rare (< 1%); and 4) the variable should cover a range of systems and domains. To ensure the inclusion of variables from a content validation process, we used a multidimensional approach [4] in which we compared the number of variables from each domain relative to the total number of variables for the following five validated FIs and/or multidimensional prognostic tools: the CSHA-FI^70^ (Original Canadian Study of Health and Aging–Frailty Index) [12], the CSHA-FI^40^ (Searle version of Canadian Study of Health and Aging–Frailty Index) [15], the FI-CGA [23], the SHARE-FI (Survey of Health, Ageing and Retirement in Europe-Frailty Index) [41], and the MPI (Multidimensional Prognostic Index) [42] (Additional file 1). It is also advisable that the proportion of missing data be < 5% [43].

Theoretically, all FIs should include a minimum number of functional deficits (ranging from 30 to 40) to maintain their predictive capacity [15]; however, by selecting variables according to severity and progression criteria [33] and grouping some of these together (especially on the functional and cognitive domains), we were able to limit the Frail-VIG index to only 22 questions assessing 25 deficits. Like other FIs, the Frail-VIG index is obtained by dividing the accumulated deficits by the total number of potential deficits, for a score ranging from 0.0 (no deficits) to 1.0 (all possible deficits).

Other requirements for the Frail-VIG index was that it meet or exceed the characteristics of existing FIs [4, 16] in terms of the submaximal limit for the FI score (99% of individuals with an FI < 0.7) [16, 22] and the asymmetric distribution density of FI scores [44], and that the score be closely correlated with mortality [14, 17].

#### Predictive validity

In order to demonstrate the predictive validity of the index, the study design included a two-year follow-up period. We determined final outcomes (living, deceased, or lost to follow up) at month 24 using only data information systems via the Shared Medical Record in Catalonia (HC3) [45], an electronic database accessible to all healthcare providers in Catalonia that allows for healthcare professionals to reliably determine whether the patient is “active” (alive) or deceased (including date of death).

### Variables and data sources

The variables included in Frail-VIG index are shown in Table 1. These variables evaluate the following domains: money, telephone and medication management—all of which are common instrumental activities of daily living (ADL); weight loss ≥5% in the last 6 months is considered a marker of nutritional worsening; emotional markers include the presence of depressive syndrome or insomnia/anxiety; the subjective perception of social vulnerability by the health care team is considered an indicator for the social domain. The following geriatric syndromes are assessed: delirium, falls, polypharmacy, and dysphagia. Symptoms with severity criteria include pain and dyspnea. Finally, the presence of chronic diseases (cancer, respiratory, cardiac, neurological, digestive, and renal disease) are also recorded.

**Table 1:** Description and characteristics of the Frail-VIG index

Domain		Variable	Description		Points
Functional	IADLs	Money management	*Needs help managing financial matters (bank, shops, restaurants)*	Yes	1
				No	0
		Telephone use	*Needs help using the telephone*	Yes	1
				No	0
		Medication management	*Needs assistance in preparing or administering medications*	Yes	1
				No	0
	ADLs	Barthel index (BI)	*No dependency (BI ≥ 95)*		0
			*Mild-moderate dependency (BI 90–65)*		1
			*Moderate-severe dependency (BI 60–25)*		2
			*Absolute dependency (BI ≤ 20)*		3
Nutritional		Malnutrition	*Weight loss ≥ 5% in the last 6 months*	Yes	1
				No	0
Cognitive		Degree of cognitive impairment	*No cognitive impairment*		0
			*Mild-moderate cognitive impairment (equivalent to GDS ≤ 5)*		1
			*Severe-very severe cognitive impairment (equivalent to GDS ≥ 6)*		2
Emotional		Depressive syndrome	*Need for antidepressant medication*	Yes	1
				No	0
		Insomnia/anxiety	*Frequent need for benzodiazepines or other psychiatric drugs with a sedative effect for insomnia/anxiety*	Yes	1
				No	0
Social		Social vulnerability	*Do health care professionals perceive the presence of social vulnerability?*	Yes	1
				No	0
Geriatric syndromes		Delirium	*Presence of delirium and/or behaviour disorder requiring antipsychotic drugs in the last* *6 months.*	Yes	1
				No	0
		Falls	*In the last* *6 months* *, ≥2 falls or hospitalization due to a fall.*	Yes	1
				No	0
		Ulcers	*Presence of ulcer (pressure or vascular, any grade)*	Yes	1
				No	0
		Polypharmacy	*Taking ≥ 5 drugs*	Yes	1
				No	0
		Dysphagia	*Difficulty swallowing when eating or drinking? Presence of aspiration respiratory infections during the last* *6 months* *?*	Yes	1
				No	0
Severe symptoms		Pain	*Need for ≥ 2 conventional analgesics and/or strong opioids for pain control*	Yes	1
				No	0
		Dyspnea	*Basal dyspnea impeding the ability to leave the house and/or opioids are frequently needed*	Yes	1
				No	0
Diseases (+)		Cancer	*Active cancer*	Yes	1
				No	0
		Respiratory	*Presence of any type of chronic respiratory disease (COPD, restrictive lung disease...)*	Yes	1
				No	0
		Cardiac	*Presence of any type of chronic heart disease (heart failure, ischemic cardiomyopathy, arrhythmia)*	Yes	1
				No	0
		Neurological	*Presence of any type of neurodegenerative disease (Parkinson, ALS,...) or a history of stroke (ischemic or hemorrhagic).*	Yes	1
				No	0
		Digestive	*Presence of any type of chronic digestive disease (chronic liver disease, cirrhosis, chronic pancreatitis, inflammatory bowel disease,…)*	Yes	1
				No	0
		Renal	*Presence of chronic renal failure (GFR < 60)*	Yes	1
				No	0
			Frail-VIG index =		

*ADLs* Basic Activities of Daily Living, *IAVDs* Instrumental Activities of Daily Living, *ALS* amyotrophic lateral sclerosis. *COPD* Chronic Obstructive Pulmonary Disease. *GFR* Glomerular Filtration Rate, *GDS* Global Deterioration Scale. (+) two point are scored if the patient presents criteria for advanced chronic illness on the NECPAL test (Annex 2; available at: http://ico.gencat.cat/web/.content/minisite/ico/professionals/documents/qualy/arxius/NECPAL-3.0-ENGLISH_full-version.pdf)

In the calculation of the Frail-VIG, binary variables are scored as “0 points” (no deficit) or “1 point” (presence of the deficit), with the exception of advanced chronic illness (defined according to the NECPAL criteria; see Additional file 2), which was scored as 2 points. For ordinal variables, the Frail-VIG uses the well-accepted cut-off points used in routine clinical practice for the Barthel Index (BI), which is used to assess basic ADLs, categorized according to the Saha criteria [46] and Reisberg’s Global Deterioration Scale (GDS), which assesses cognitive impairment, categorizing patients into one of 3 groups [47].

To prevent intercurrent illnesses (that is, the conditions that led to hospitalization) from influencing the Frail-VIG score, the index score identifies only the patient’s baseline condition (that is, ≥ 1 month before hospitalization and/or prior to onset of the clinical process leading to hospital admission). This information is collected on the patient’s first day in the hospital by interview (patient and/or family) to obtain a full medical history and/or by checking the medical record to determine current prescriptions.

### Statistical methods

Descriptive statistics of the variables were calculated using the SPSS software program (IBM; Chicago, IL; USA). Statistical significance (*p* < 0.05) with 95% confidence intervals was obtained by comparing mean values (for quantitative variables) and percentages (for qualitative variables).

Survival estimates were obtained for the 24-month follow-up. The survival analysis was performed using the Survival, pROC, and RMS packages from the R project (https://www.r-project.org). Survival curves were computed according to the Frail-VIG index scores, grouping these according to predefined criteria before the analysis into seven interval ranges (0–0.15, 0.16–0.25, 0.26–0.35, 0.36–0.45, 0.46–0.55, 0.56–0.65, and 0.66–1), and then the survival curves were compared via the log-rank test. The ROC curves were analysed to determine the predictive capacity of the Frail-VIG index.

## Results

### Cohort description and analysis of the variables

A total of 590 patients were included. Mean (standard deviation; SD) patient age was 86.4 (5.6) years. Women comprised 57.5% (*n* = 339) of the sample. Most (83.9%) of the patients admitted to the AGU were frail (Frail-VIG index score > 0.25). Fifty-three patients (8.9%) died during hospitalization. No missing values were observed for any of the study variables. Table 2 shows the descriptive results for the study variables.

**Table 2:** Descriptive results for the whole sample and comparative outcomes between surviving vs. non-surviving patients

Variable		Total (*n* = 590)	*Death* during the 24-month follow up		*p*-value
			Yes (*n* = 338)	No (*n* = 252)	
Mean age (years)		86.39 *(+/−5.58)*	86.55 *(+/−5.76)*	86.17 *(+/−5.34)*	0.420
Sex	Men	251 *(42.5%)*	160 *(63.7%)*	91 *(36.3%)*	0.006
	Women	339 *(57.5%)*	178 *(52.5%)*	161 *(47.5%)*	
Advanced chronic disease	Yes	260 *(44.1%)*	220 *(84.6%)*	40*(15.4%))*	< 0.001
	No	330 *(55.9%)*	118 *(35.8%)*	212 *(64.25%)*	
Length of hospital stay	Mean days	5.88 *(+/−3.26)*	5.88*(+/−3.36)*	5.33*(+/−3.12)*	0.982
Destination after discharge	Home	135 *(22.9%)*	59 *(43.7%)*	76 *(56.3%)*	< 0.001
	Residence	81 *(13.7%)*	45 *(55.6%)*	36 *(44.4%)*	
	Intermediate care facility	321 *(54.4%)*	181 *(56.4%)*	140 *(43.6%)*	
	Death	53 *(9%)*			
IADLs	(0–1–2-3)	0.85 (+/−1.15)	0.53	1.27	< 0.001
ADLs	Mean Barthel index	62.81 (+/−29.71)	57.26	70.24	< 0.001
Malnurition		190 *(32.2%)*	175 *(92.1%)*	15 *(7.9%)*	< 0.001
Cognitive Impairment	None	231 *(39.2%)*	112 *(48.5%)*	119 *(51.5%)*	< 0.001
	Mild/Moderate	257 *(43.6%)*	154 *(59.9%)*	103 *(40.1%)*	
	Severe	102 *(17.3%)*	72 *(70.6%)*	30 *(29.4%)*	
Emotional Status	Euthymic mood	306 *(51.9%)*	175 *(57.2%)*	131 *(42.8%)*	0.166
	Depressive syndrome	251 *(42.5%)*	139 *(55.4%)*	112 *(44.6%)*	
	Not evaluable	33 *(5.6%)*	24 *(72.7%)*	9 *(27.3%)*	
	Insomnia	337 *(63.9%)*	214 *(63.5%)*	123 *(36.5%)*	< 0.001
Social Vulnerability		19 *(3.2%)*	14 *(73.7%)*	5 *(26.3%)*	0.142
Geriatric Syndromes	Delirium	334 *(56.6%)*	220 *(65.9%)*	114 *(34.1%)*	< 0.001
	Falls	348 *(59%)*	197 *(56.6%)*	151 *(43.4%)*	0.689
	Ulcers	76 *(12.9%)*	64 *(84.2%)*	12 *(15.8%)*	< 0.001
	Polypharmacy	474 *(80.3%)*	288 *(60.8%)*	186 *(39.2%)*	0.001
	Dysphagia	255 *(43.2%)*	205 *(80.4%)*	50 *(19.6%)*	< 0.001
Severe Symptoms	Pain	146 *(24.7%)*	103 *(70.5%)*	43 *(29.5%)*	< 0.001
	Dyspnea	69 *(11.7%)*	59 *(85.5%)*	10 *(14.5%)*	< 0.001
Chronic Diseases	Cancer	87 *(14.7%)*	73 *(83.9%)*	14 *(16.1%)*	< 0.001
	Respiratory	164 *(27.8%)*	107 *(65.2%)*	57 *(34.8%)*	0.015
	Cardiac	352 *(59.7%)*	233 *(66.2%)*	119 *(33.8%)*	< 0.001
	Neurological	155 *(26.8%)*	93 *(60.0%)*	62 *(40.0%)*	0.427
	Digestive	68 *(11.5%)*	53 *(77.9%)*	15 *(22.1%)*	< 0.001
	Renal	291 *(49.3%)*	208 *(71.5%)*	83 *(28.5%)*	< 0.001

*ADLs* Basic Activities of Daily Living, *IADLs* Instrumental Activities of Daily Living

### Variable selection and results of construct evaluation

The deficits included in the Frail-VIG index are those commonly associated with age and adverse health outcomes and these deficits were carefully selected to represent the main domains to be assessed (see Additional file 1).

**Fig. 1:**
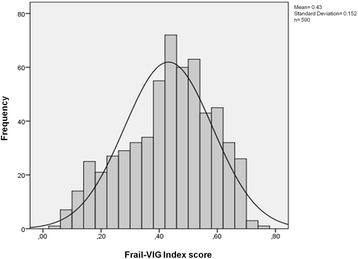
Distribution of Frail-VIG index scores at baseline (*n* = 590)

The prevalence rate for each variable was < 80% (except for polypharmacy, which was exactly 80%) and none could be considered rare (< 1%). Although the prevalence for some variables—for example, polypharmacy—was high, none of these variables are universal, even in 85-year old patients [48]. The overall construct validation process [4] shows that the distribution of variables by domain (as a percentage of the overall index) was similar to other validated FIs (see Additional file 1). Despite the high mean age of this population cohort and the large percentage of individuals presenting with advanced chronic conditions, the submaximal limit (0.7) for FI scores was maintained (that is, the Frail-VIG index was < 0.7 in 99.3% of patients). The distribution of scores on the Frail-VIG index tended towards asymmetry (asymmetry coefficient, 0.37) (Fig. 1).

### Mortality at 24 months

**Fig. 2:**
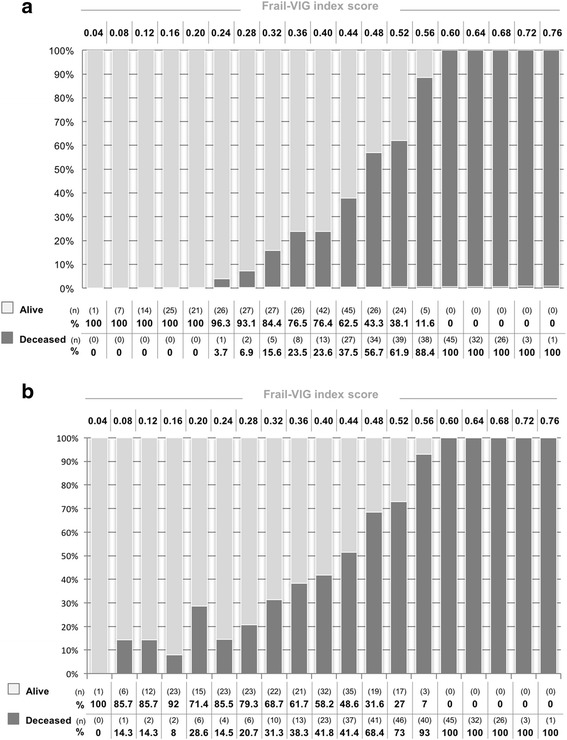
Distribution in number (n) and percentage (%) of surviving and non-surviving patients at 12 (panel **a**) and 24 (panel **b**) months according to the Frail-VIG index score

The cohort was followed for 24 months or until death, whichever came first. At 24 months, the mortality rate was 57.3% (*n* = 338). None of the patients were lost to follow up. Table 2 shows differences in mortality rates for each individual study variable. The mortality rate increased progressively in line with higher Frail-VIG scores (Fig. 2), especially at the 12 month follow up (Fig. 2; panel A).

**Fig. 3:**
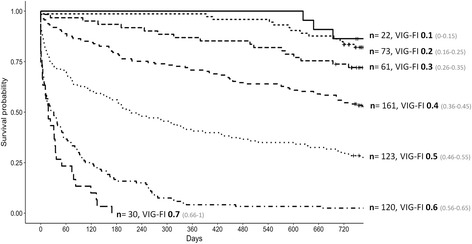
Kaplan–Meier curves according to Frail-VIG index score

We evaluated the correlation between mortality and the Frail-VIG index by means of the log-rank test (Fig. 3)*,* comparing the seven different survival curves based on the score intervals described in the statistical analysis section above. Highly significant differences were observed between the various range intervals (*X*^2^ = 433.4, *p* < 0.0001). Table 3 shows the number of individuals in each frailty index category who had died at 24 months of follow-up.

**Table 3:** People alive and deceased at 12 and 24 months follow-up in each frailty index category

Frail-VIG index category		0.1 *(0–0.15)*	0.2 *(0.16–0.25)*	0.3 *(0.26–0.35)*	0.4 *(0.36–0.45)*	0.5 *(0.46–0.55)*	0.6 *(0.56–0.65)*	0.7 *(0.66–1)*	Total
*12 months Follow-up*	Alive	22	72	54	113	50	5	0	316
	Deceased	0	1	7	48	73	115	30	274
	*Deceased (%)*	*0*	*1.4*	*11.4*	*45.3*	*70.7*	*97.5*	*100*	*46.4*
*24 months Follow-up*	Alive	19	61	45	88	36	3	0	252
	Deceased	3	12	16	73	87	117	30	338
	*Deceased (%)*	*13.6*	*16.4*	*26.2*	*45.3*	*70.7*	*97.5*	*100*	*57.3*
Total		22	73	61	161	123	120	30	590

On the ROC curve analysis, the area under the curve (AUC) values at 3, 6, 9, 12 and 24 months were, respectively, as follows: 0.87 (range, 0.84–0.90), 0.88 (0.85–0.91), 0.89 (0.87–0.92), 0.9 (0.88–0.92), and 0.85 (0.82–0.88) (Additional file 3). The optimal cut-off point was 0.46 in all cases, revealing a better performance (as measured by the Youden index) at 12 months (0.62), with a sensitivity and specificity of 0.80 and 0.83, respectively (Additional file 4).

According to the participating clinicians, administration time for the Frail-VIG index ranged from 5 to 10 min.

## Discussion

### Key results

At the 24 month follow-up, we found statistically significant differences (*p* < 0.05) between the two groups (living vs. deceased) for most of the Frail-VIG variables, a finding that confirms the index’s discriminative capacity. The only variables that were not statistically significant were 1) social vulnerability—likely due to the limited number of patients with social vulnerability (19 patients; 3.2%), 2) emotional status (although there were significant differences [p 0.016] at the 12-month follow up), and 3) falls (probably because we did not differentiate between severe and non-severe falls).

These results, which met the previously-specified internal validation criteria, support the validity of the Frail-VIG index [16, 44]**.** All criteria were fully met, both in terms of the variables included and with regard to the construct’s validation criteria.

The strong correlation between the Frail-VIG index score and mortality was consistent with published data [14, 17]. The 12- and 24-month AUC were 0.90 and 0.85, respectively, demonstrating that the Frail-VIG index offers a better prognostic capacity than other previously published FIs (whose AUCs range from 0.6 to 0.8) [4, 14, 49, 50]. A study conducted by Pilotto et al. evaluated several different FIs [51] in a group of hospitalized patients (20 Italian geriatric units; *n* = 2033), reporting a 12-month AUC of 0.69 for the FI-SOF (Frailty Index-Study of Osteoporotic Fractures) [52], 0.73 for the **CSHA-FI**^**70**^, 0.73 for the FI-CGA, and 0.75 for the MPI. A study by Ritt et al. involving 307 hospitalized inpatients in an AGU in Erlangen (Germany) compared the predictive capacity of 3 different FIs based on CGA, finding an AUC at 6 months that ranged from 0.77 to 0.84 [53].

Although the excellent predictive capacity of the Frail-VIG index may seem surprising, we believe that the following factors may have positively influenced our results: (1) The characteristics of the instrument itself, which incorporates only those variables that are routinely used in conventional CGAs. Moreover, most of these variables are good predictors of mortality, as evidenced by the statistically significant differences between the two groups (alive vs. deceased) for practically all of the individual variables included in the index (Table 2). (2) There were no missing data from the baseline data due to the systematic data collection processes during routine clinical practice of the health care team. Similarly, no data were missing during follow up thanks to the reliability of the “Shared Medical Record” system in Catalonia (HC3). (3) The instrument was administered by a small group of professionals with extensive expertise in geriatric assessment. Although the questionnaire is both short and simple (containing only basic questions with dichotomous response options), we do not know if administration of this instrument by untrained staff or in other settings could lead to different results; nor do we have any data on possible interobserver variability. (4) We cannot rule out the possibility of selection bias caused by the AGU admission criteria, which could have led us to select a population with high mortality rates. (5) The intercurrent process that led to hospital admission may have affected the final mortality results, potentially increasing specifically the mortality rate in those patients who were the most frail prior to admission.

When we compared the results at the 12 and 24 month follow up periods, we found that the prognostic accuracy of the index tends to decrease over time (Fig. 3, Table 3 and Additional files 3 and 4). The most plausible explanation for this finding is the dynamic nature of frailty [54]: the Frail-VIG index score for each patient reflects a static vision of reality; that is, it indicates the patient’s status only at the time of data collection. However, given the patient profile of this cohort (elderly patients with high multimorbidity rates), it is logical to expect that these patients will continue to accumulate deficits during the follow up period, thus increasing their Frail-VIG index score; this would also explain 24 month mortality in individuals whose initial Frail-VIG score (< 0.25) did not indicate the presence of frailty.

Administration of the Frail-VIG index does not preclude the use of the CGA for systematic multidimensional assessment [55], but instead offers the advantage of a RGA: only 5–10 min of time are needed to complete the Frail-VIG; by contrast, the CSHA-FI^70^ [12] requires around 20–30 min [21] and the FI-CGA [23] about 25 min [56].

### Utility of the frail-VIG index

**Fig. 4:**
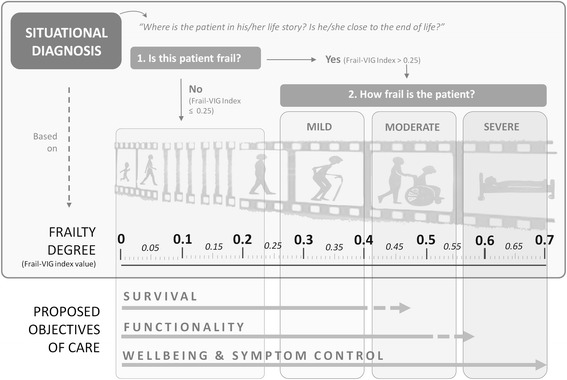
Conceptual Model of Situational Diagnosis (based on degree of frailty) and the proposed care objectives

Although more studies will be needed to confirm the benefits of using FIs to assess geriatric patients, we believe the Frail-VIG index to be a valuable tool:

1. The index can accurately discriminate between frail and non-frail patients. The fact that more than 80% of the patients admitted to the AGU—a unit specifically designed for frail patients—are, in fact, frail, supports the appropriateness of the unit’s admission criteria.
2. The Frail-VIG index score assesses the degree of frailty as a continuous variable. This can help clinicians make an accurate situational diagnosis [31]: the ability to determine the patient’s approximate biological age (degree of frailty) relative to his/her chronological age, can be particularly relevant when selecting or adjusting the treatment to develop an appropriate therapeutic plan. For instance, identifying advanced, progressive frailty (after potentially reversible causes have been ruled out), helps in analysing the risk/benefit of aggressive interventions. Conversely, assessing the degree of frailty can also be useful in decision-making for patients at risk of undertreatment who, though having fair reserve, could be denied additional diagnostic efforts or specific therapeutic interventions due to age. Frailty assessment using FIs allow us to reduce uncertainty by providing better understanding on the patient’s overall situation, helping to improve the dialogue between the patient, family and health care professionals in regards to expectations and objectives in the shared decision-making process (Fig. 4).
3. Related to the previous point, quantification of the degree of frailty by means of the Frail-VIG index could facilitate the creation of a common reference for communication with health care professionals in other specialities. In addition, this could potentially allow clinicians to monitor the results of any interventions. Finally, quantification could be highly valuable in patient care and for research purposes [18].

### Study limitations and next steps

The data described here should be interpreted with caution given that our cohort is not representative of the general population. An important limitation of the present study is that the Frail-VIG index has not been validated by comparing it to other instruments in the same cohort, nor compared to other variables (for example, institutionalization or use of resources). Additional studies are required to assess the reliability and reproducibility of this index in other settings and populations. Some study variables were obtained by reviewing the patient’s medications without confirming the suitability of the prescription and/or dose for the patient’s condition; this approach to obtaining certain variables may have biased the study outcomes. Assessment of certain variables (for example, social needs) needs to be improved to increase the reliability and accuracy of the tool. Finally, although the professionals agreed about the time needed to administer the instrument (that is, more than 5 min but less than 10 min), we did not systematically measure (using a timer) these data.

Finally, the next steps in the development of the Frail-VIG index are the following: (1) validate the instrument in an independent cohort, in other settings and distinct populations; (2) demonstrate its applicability and utility for professionals in routine clinical practice; and (3) as with most FIs, the true challenge is to demonstrate whether the routine, systematic use of this tool actually improves the health outcomes of the patients in the “triple aim” framework [57].

## Conclusions

The Frail-VIG index was developed to facilitate frailty assessment during routine clinical practice in order to improve the management of at-risk patients. The main benefits of the Frail-VIG index are its simplicity—22 simple, dichotomous questions to assess 25 different deficits—and short administration time (5 to 10 min), which allows physicians’ to incorporate frailty assessment into the overall evaluation of elderly patients. Perhaps more importantly, the Frail-VIG index can reliably discriminate between different degrees of frailty (up to seven categories in the present study) and can accurately predict 12- and 24-month mortality. The results of the current study support the use of the Frail-VIG index in routine clinical practice. Additional research is needed to confirm its validity in a wider range of populations.

## Additional files

Additional file 1: Percentage of variables by domain versus the other four validated frailty indices. The distribution of variables by domain (as a percentage of the overall index) was similar to other validated FIs: the Original Canadian Study of Health and Aging – Frailty Index (CSHA-FI^70^), the Searle version of Canadian Study of Health and Aging – Frailty Index (CSHA-FI^40^), the Frailty Index based on Comprehensive Geriatric Assessment (FI-CGA) and the SHARE-Frailty Index (SHARE-FI). However, the Frail-VIG showed a higher weighting of geriatric syndromes and symptoms and a lower weighting for the functional domain; it should be noted that the Frail-VIG is the only index to include the social domain. (DOCX 14 kb)

Additional file 2: Specific NECPAL severity criteria / progression / advanced disease. Severity and/or progression criteria for advanced chronic illness (oncological, pulmonary, cardiovascular, neurological, hepatobiliary-digestive, kidney) defined in the NECPAL test. Abbreviations: FEV1: forced expiratory volume in 1 s. VC: Vital Capacity; DLCO: Diffusion capacity for carbon monoxide; NYHA: New York Hearth Association. EF: Ejection Fraction; PAH: Pulmonary Arterial Hypertension; PAPs: pulmonary artery pressure; GFR: glomerular filtration rate; MS: multiple sclerosis; ALS: amyotrophic lateral sclerosis. (DOCX 15 kb)

Additional file 3: ROC curves. (Panel A) Area Under the Curve (AUC) at 3, 6, 9, 12, and 24 months. (Panel B) Changes in the AUC over the course of the follow-up period. This shows how the AUC tends to increase up to month 12 after which the prognostic accuracy begins to decline until month 24. (TIFF 700 kb)

Additional file 4: Area Under the Curve (AUC), optimal cut-off point of the Frail-VIG index related to the sensitivity and specificity and Youden’s index at 3, 6, 9, 12, and 24 months. The optimal cut-off point is 0.46 in all cases, with the best performance according to Youden’s index observed at 12 months (0.62), with a sensitivity and specificity of 0.80 and 0.83, respectively. (DOCX 14 kb)

## Abbreviations

ADLs: Basic Activities of Daily Living
AGU: Acute Geriatric Unit
ALS: amyotrophic lateral sclerosis
AUC: Area Under the Curve
CGA: Comprehensive Geriatric Assessment
COPD: Chronic Obstructive Pulmonary Disease
CSHA-FI^40^: Searle version of Canadian Study of Health and Aging – Frailty Index
CSHA-FI^70^: Original Canadian Study of Health and Aging – Frailty Index
FI: Frailty Index
FI-CGA: Frailty Index based on Comprehensive Geriatric Assessment
FI-SOF: Frailty Index-Study of Osteoporotic Fractures
GDS: Global Deterioration Scale
GFR: Glomerular Filtration Rate
IAVDs: Instrumental Activities of Daily Living
MPI: Multidimensional Prognostic Index
RGA: Rapid Geriatric Assessment
SHARE-FI: Survey of Health, Ageing and Retirement in Europe-Frailty Index
