# Supplementary material for: Frail-VIG index: a concise frailty evaluation tool for rapid geriatric assessment
Source: BMC Geriatr. 2018 Jan 26;18:29. doi: 10.1186/s12877-018-0718-2 (PMC5787254; doi:10.1186/s12877-018-0718-2)
Supplement: Supplementary file 1 — Percentage of variables by domain versus the other four validated frailty indices. The distribution of variables by domain (as a percentage of the overall index) was similar to other validated FIs: the Original Canadian Study of Health and Aging – Frailty Index (CSHA-FI70), the Searle version of Canadian Study of Health and Aging – Frailty Index (CSHA-FI40), the Frailty Index based on Comprehensive Geriatric Assessment (FI-CGA) and the SHARE-Frailty Index (SHARE-FI). However, the Frail-VIG showed a higher weighting of geriatric syndromes and symptoms and a lower weighting for the functional domain; it should be noted that the Frail-VIG is the only index to include the social domain. (DOCX 14 kb) [file 12877_2018_718_MOESM1_ESM.docx]

**Additional file 1:** Percentage of variables by domain versus the other four validated frailty indices & multidimensional prognostic tools**.**

|  | | FRAILTY INDICES / MULTIDIMENSIONAL PROGNOSTIC TOOLS | | | | | |
| --- | --- | --- | --- | --- | --- | --- | --- |
| DOMAIN | | **CSHA-FI^70^** | **CSHA-FI^40^** | **FI-CGA** | **SHARE-FI^40^** | **MPI** | **Frail-VIG index** |
| Functional | IADL | n=4 (6 %) | n=5 (12.5 %) | n=6 (12 %) | n=5 (12.5 %) | n=8 (12.7 %) | n=3 (12 %) |
|  | ADL | n=7 (10 %) | n=10 (25 %) | n=9 (17 %) | n=9 (22.5 %) | n=6 (9.5 %) | n=3 (12 %) |
| Nutritional | | - | n=2 (5 %) | n=1 (2 %) | n=2 (5 %) | n=18 (28.5%) | n=1 (3 %) |
| Cognitive | | n=9 (13 %) | n=1 (2.5 %) | n=1 (2 %) | n=1 (2.5 %) | n=10 (15.9 %) | n=3 (12 %) |
| Emotional | | n=7 (10 %) | n=3 (7.5 %) | n=2 (4 %) | n=3 (7.5 %) | - | n=2 (8 %) |
| Social | | - | - | - | - | n=1 (1.6 %) | n=1 (3 %) |
| Geriatric Syndromes | | n=5 (7 %) | - | n=7 (13 %) | n=2 (5 %) | n=1 (1.6 %) | n=4 (16 %) |
| Symptoms | | - | - | n=1 (2 %) | n=1 (2.5 %) | - | n=2 (8 %) |
| Chronic diseases & comorbidity | | n=15 (21 %) | n=8 (20 %) | n=11 (21 %) | n=10 (25 %) | n=14 (22.2 %) | n=6 (26 %) |
| Others | | n=23 (33 %) | n=11 (27.5 %) | n=14 (27 %) | n=7 (17.5 %) | n=5(8 %) | - |
| TOTAL DEFICITS | | x/70 | x/40 | x/52 | x/40 | x/63 | x/25 |

**ADLs:** Activities of Daily Living. **IADLs**: Instrumental Activities of Daily Living. **CSHA-FI^70^**: Original Canadian Study of Health and Aging – Frailty Index. **CSHA-FI^40^**: Searle version of Canadian Study of Health and Aging – Frailty Index. **FI-CGA**: Frailty Index –Comprehensive Geriatric Assessment. **MPI**: Multidimensional Prognostic Index. **SHARE-FI**: SHARE-Frailty Index.
