# Supplementary material for: Frail-VIG index: a concise frailty evaluation tool for rapid geriatric assessment
Source: BMC Geriatr. 2018 Jan 26;18:29. doi: 10.1186/s12877-018-0718-2 (PMC5787254; doi:10.1186/s12877-018-0718-2)
Supplement: Supplementary file 2 — Specific NECPAL severity criteria / progression / advanced disease. Severity and/or progression criteria for advanced chronic illness (oncological, pulmonary, cardiovascular, neurological, hepatobiliary-digestive, kidney) defined in the NECPAL test. Abbreviations: FEV1: forced expiratory volume in 1 s. VC: Vital Capacity; DLCO: Diffusion capacity for carbon monoxide; NYHA: New York Hearth Association. EF: Ejection Fraction; PAH: Pulmonary Arterial Hypertension; PAPs: pulmonary artery pressure; GFR: glomerular filtration rate; MS: multiple sclerosis; ALS: amyotrophic lateral sclerosis. (DOCX 15 kb) [file 12877_2018_718_MOESM2_ESM.docx]

**Additional file 2:**  Specific NECPAL severity criteria / progression / advanced disease

| DISEASE | SPECIFIC NECPAL CRITERIA SEVERITY / PROGRESSION /  ADVANCED DISEASE |
| --- | --- |
| Cancer | - Metastatic or advanced locoregional cancer in progression - Persistent, uncontrolled or refractory symptoms despite treatment |
| Chronic Lung disease | - Shortness of breath at rest on minimal exertion - Confined to home with severe limitations - Spirometric criteria for severe obstruction (FEV1 <30%) or severe restrictive criteria (VC <40% / DLCO <40%) - Gasometric criteria for chronic oxygen therapy at home - Need for continuous corticosteroid treatment - Associated symptomatic heart failure |
| Chronic Heart disease | - Shortness of breath at rest on minimal exertion - Heart failure (NYHA stage III or IV), nonsurgical severe valvular disease, or nonsurgical advanced coronary disease - Baseline echocardiography: EF <30% or severe PAH (PAPs > 60) - Associated renal failure (GFR <30 l / min) |
| Chronic Vascular Neurological Disease (stroke) | - In acute phase (< 3 months after stroke): low consciousness state - In chronic phase (< 3 months after stroke) repeated medical complications (or severe dementia) |
| Chronic Neurological diseases: (Motor neuron, MS, ALS, Parkinson) | - Progression of functional, nutritional, and/or cognitive declines - Complex or resistant symptoms - Persistent dysphagia - Increasing communication difficulties - Frequent aspiration pneumonias, dyspnea or respiratory failure |
| Chronic Liver disease | - Advanced cirrhosis (Child class C). Refractory ascites, hepato-renal syndrome and/or upper digestive bleeding despite treatment. - Hepatic carcinoma stage C or D |
| Chronic Kidney disease | - Severe renal failure (GFR < 15) in patients not eligible for, or who refuse, transplant or dialysis - End of dialysis or transplant failure |

**FEV1**: Forced Expiratory Volume in 1 second; **VC**: Vital Capacity; **DLCO**: Diffusion Capacity for Carbon Monoxide; **NYHA**: New York Hearth Association; **EF**: Ejection Fraction; **PAH**: Pulmonary Arterial Hypertension; **PAPs**: Pulmonary Artery Pressure; **GFR**: Glomerular Filtration Rate; **MS**: Multiple Sclerosis; **ALS**: Amyotrophic Lateral Sclerosis.
