# Supplementary figures and images for: Frail-VIG index: a concise frailty evaluation tool for rapid geriatric assessment
Source: BMC Geriatr. 2018 Jan 26;18:29. doi: 10.1186/s12877-018-0718-2 (PMC5787254; doi:10.1186/s12877-018-0718-2)

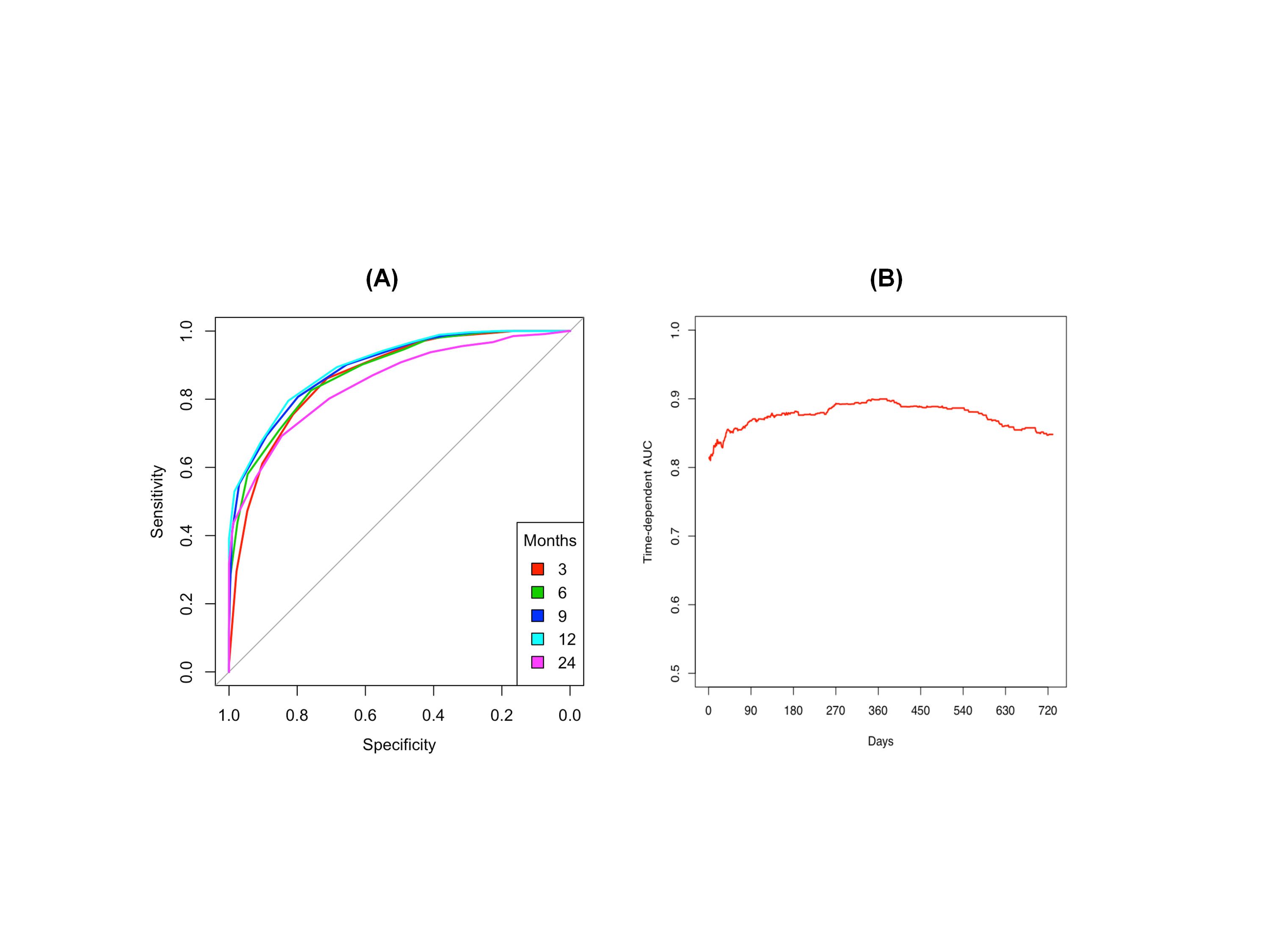

Supplement: Supplementary file 3 — ROC curves. (Panel A) Area Under the Curve (AUC) at 3, 6, 9, 12, and 24 months. (Panel B) Changes in the AUC over the course of the follow-up period. This shows how the AUC tends to increase up to month 12 after which the prognostic accuracy begins to decline until month 24. (TIFF 700 kb) [file 12877_2018_718_MOESM3_ESM.tif]
