# Supplementary material for: Frail-VIG index: a concise frailty evaluation tool for rapid geriatric assessment
Source: BMC Geriatr. 2018 Jan 26;18:29. doi: 10.1186/s12877-018-0718-2 (PMC5787254; doi:10.1186/s12877-018-0718-2)
Supplement: Supplementary file 4 — Area Under the Curve (AUC), optimal cut-off point of the Frail-VIG index related to the sensitivity and specificity and Youden’s index at 3, 6, 9, 12, and 24 months. The optimal cut-off point is 0.46 in all cases, with the best performance according to Youden’s index observed at 12 months (0.62), with a sensitivity and specificity of 0.80 and 0.83, respectively. (DOCX 14 kb) [file 12877_2018_718_MOESM4_ESM.docx]

**Additional file 4:**  Area Under the Curve, optimal cut-off point of Frail-VIG index related to sensitivity and specificity, and Youden's index at 3, 6, 9, 12, and 24 months.

| Months | 3 | 6 | 9 | 12 | 24 |
| --- | --- | --- | --- | --- | --- |
| AUC | 0.87 | 0.88 | 0.89 | 0.9 | 0.85 |
| Frail-VIG index | 0.46 | 0.46 | 0.46 | 0.46 | 0.46 |
| Sensitivity | 0.86 | 0.83 | 0.81 | 0.80 | 0.69 |
| Specificity | 0.71 | 0.76 | 0.80 | 0.83 | 0.85 |
| Youden's index | 0.57 | 0.59 | 0.60 | 0.62 | 0.54 |

**AUC**: Area Under the Curve.
